# Supplementary material for: Regulating adsorption selectivity by charge-polarized Auδ−-Cuδ+ site for stable glucose electrooxidation
Source: Nat Commun. 2026 Apr 23;17:4372. doi: 10.1038/s41467-026-72465-x (PMC13176337; doi:10.1038/s41467-026-72465-x)
Supplement: Supplementary file 1 — Supplementary Information [file 41467_2026_72465_MOESM1_ESM.pdf]

Supplementary information for

Regulating adsorption selectivity by charge-polarized $Au^{\delta-}$-$Cu^{\delta+}$ site for stable glucose electrooxidation

Yunpeng Liu[1], Xiaolong Tao[1], Chuqiang Huang[1], Kai Zhao[1], Binglu Deng[1*], Feng Peng[2*]

[1]School of Materials and Energy, Foshan University, Foshan, 528000, China

[2]School of Chemistry and Chemical Engineering, Guangzhou University, Guangzhou 510006, China

*Corresponding authors:

Foshan University, E-mail: dengbl@fosu.edu.cn (B. Deng);

Guangzhou University, E-mail: fpeng@gzhu.edu.cn (F. Peng)

Supplementary Figures

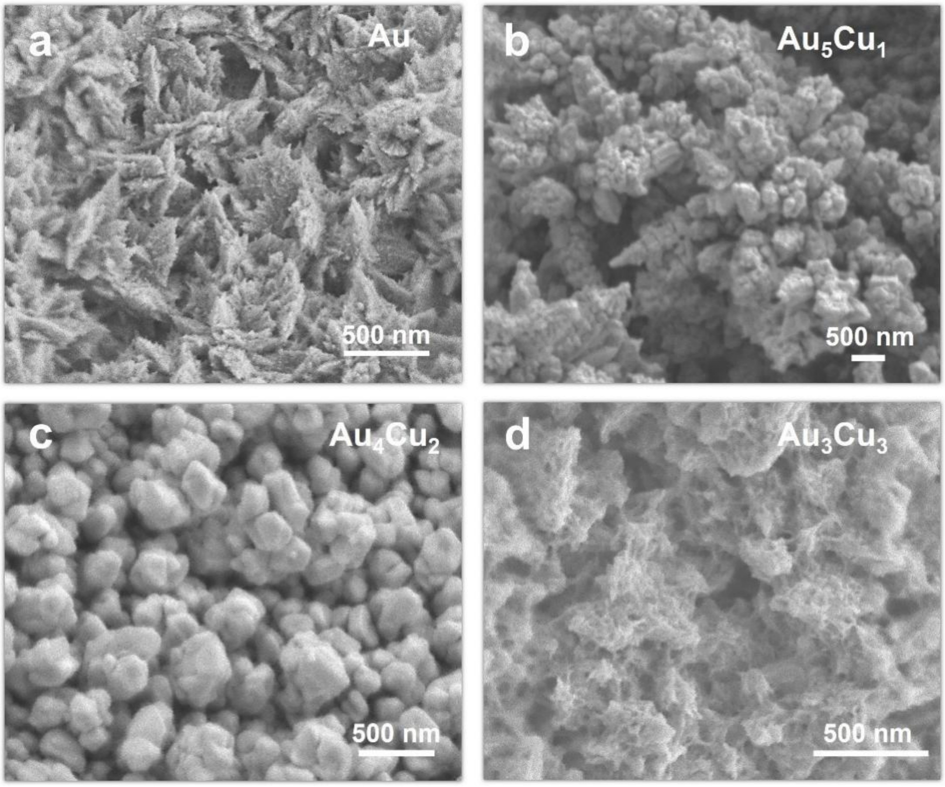

Supplementary Fig. 1. SEM images of (a) Au, (b) Au_5Cu_1, (c) Au_4Cu_2, and (d) Au_3Cu_3 samples.

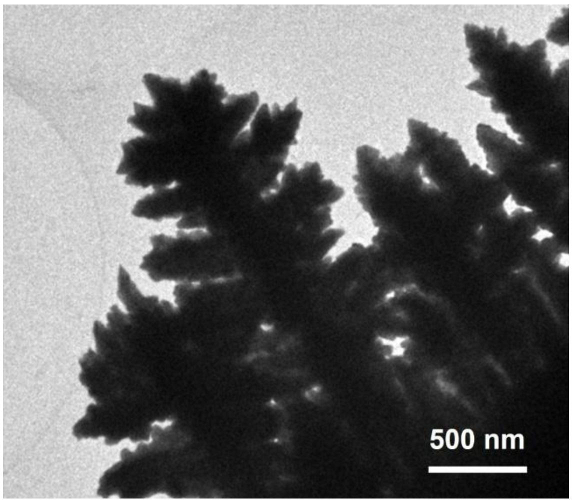

Supplementary Fig. 2. TEM image of Au.

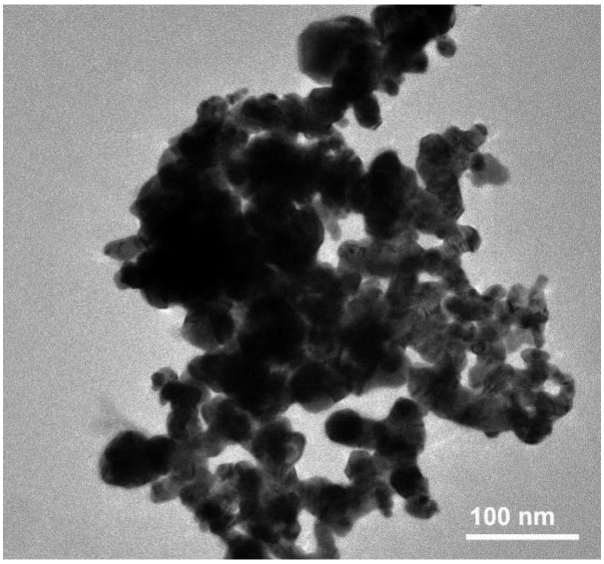

Supplementary Fig. 3. (a) TEM image of Au_4Cu_2.

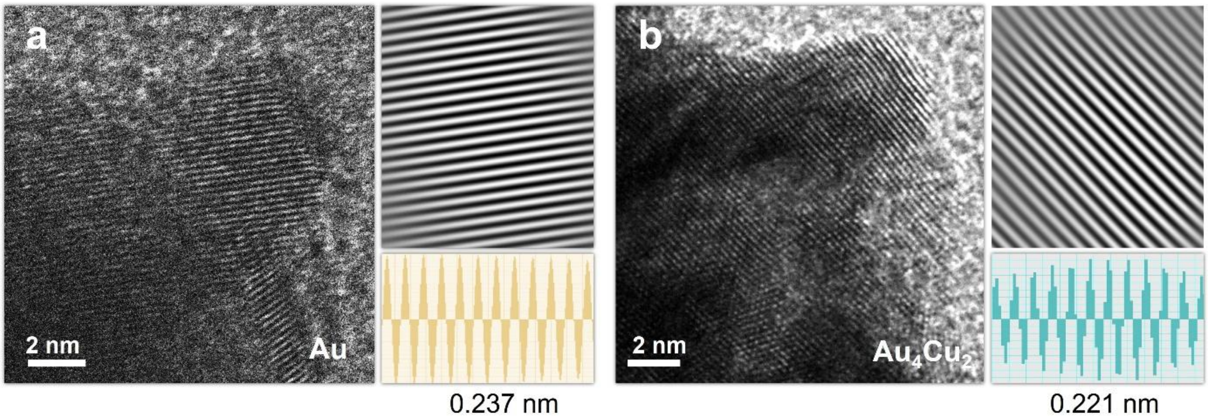

Supplementary Fig. 4. HRTEM images and lattice spacings of (a) Au and (b)Au_4Cu_2.

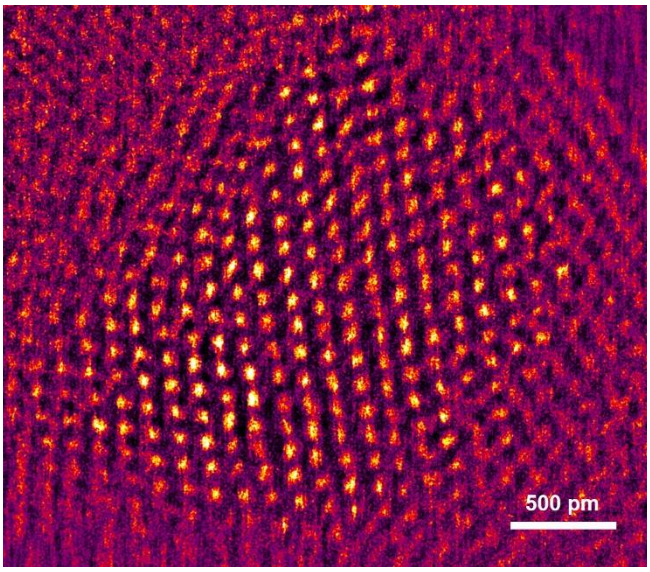

Supplementary Fig. 5. Atomic-resolution HAADF-STEM image of Au_4Cu_2.

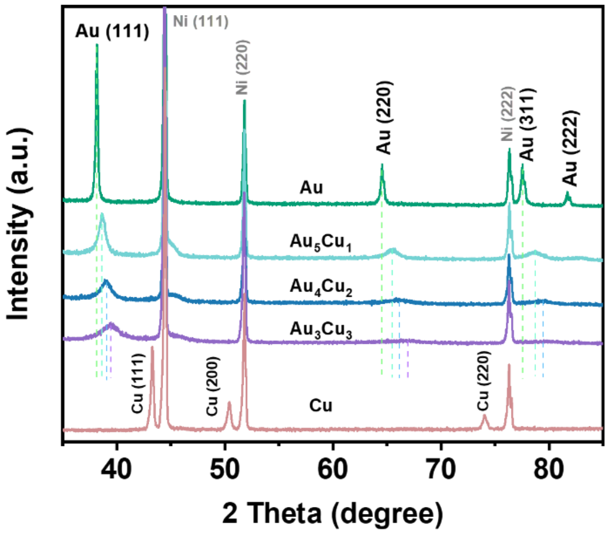

Supplementary Fig. 6. XRD patterns of different samples.

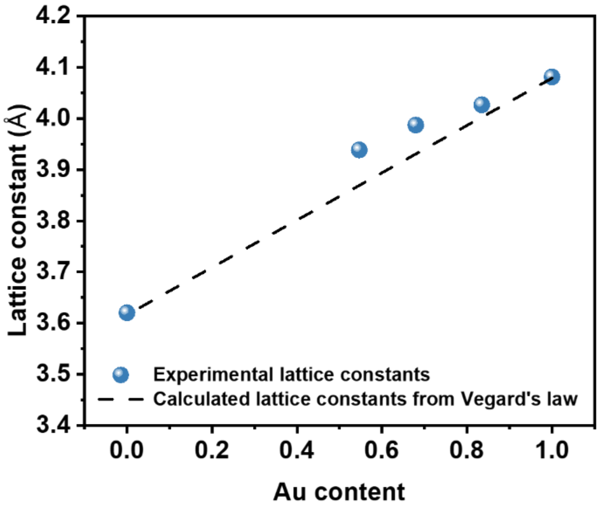

Supplementary Fig. 7. Calculated and experimental lattice constants for the AuCu alloys. Calculated lattice constants are obtained from Vegard's law.

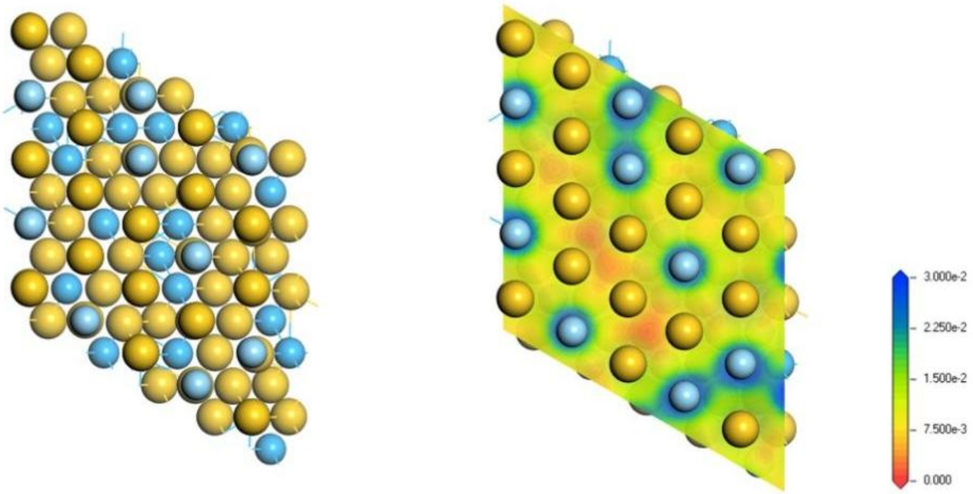

Supplementary Fig. 8. Differential charge density of Au_4Cu_2,

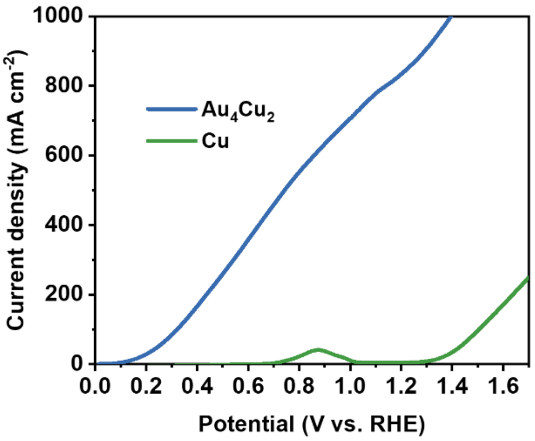

Supplementary Fig. 9. LSV profiles of Au_4Cu_2 and Au electrocatalysts for glucose oxidation in 1.0 M KOH with 0.2 M glucose.

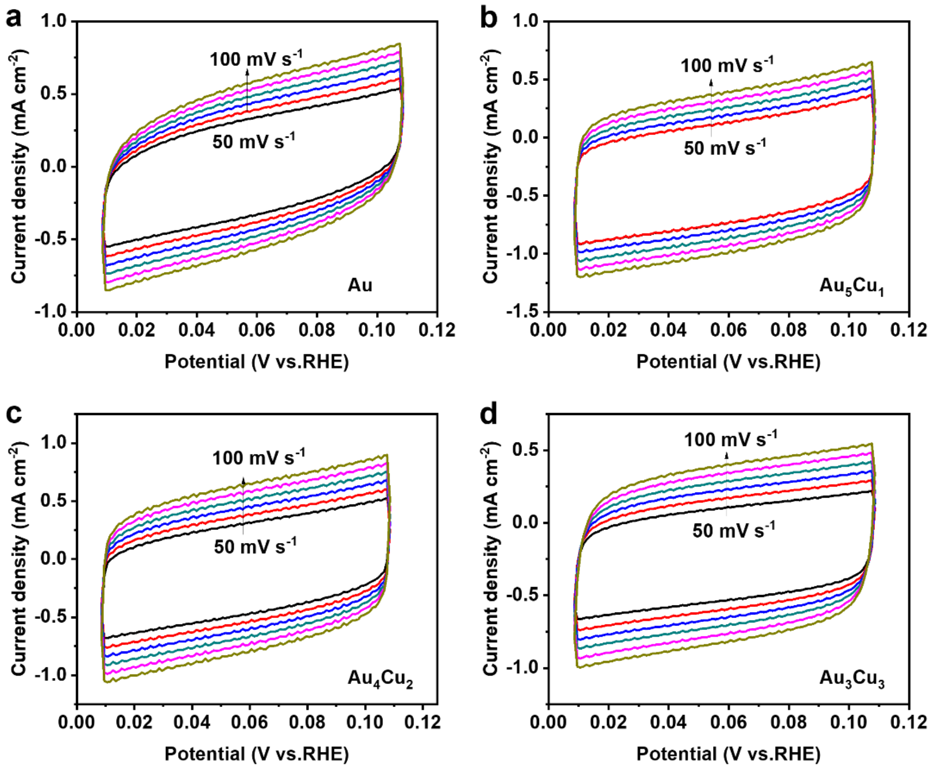

Supplementary Fig. 10. CV curves of (a) Au, (b) Au_5Cu_1, (c) Au_4Cu_2 and (d) Au_3Cu_3 (after LSV tests of GOR) at different scan rates from 50 to 100 mV s^{-1} in 1 M KOH with 0.2 M glucose.

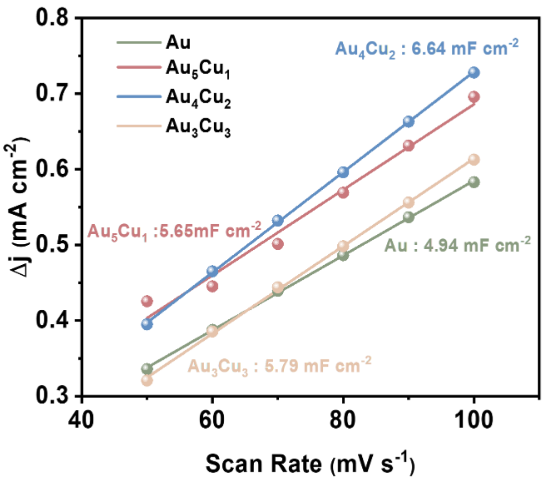

Supplementary Fig. 11. The extracted double-layer capacitances (C_{dl}) of different electrodes using a CV method after LSV tests of GOR.

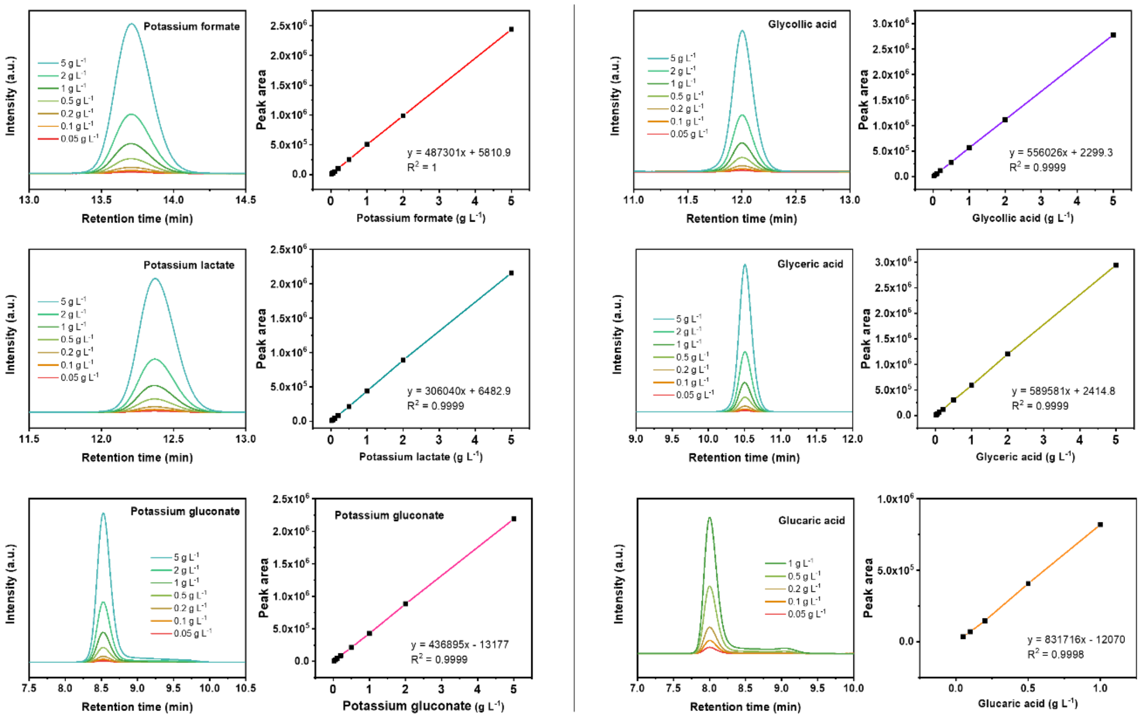

Supplementary Fig. 12. The HPLC spectra of the standard substances and the corresponding calibration curves established with the external standards.

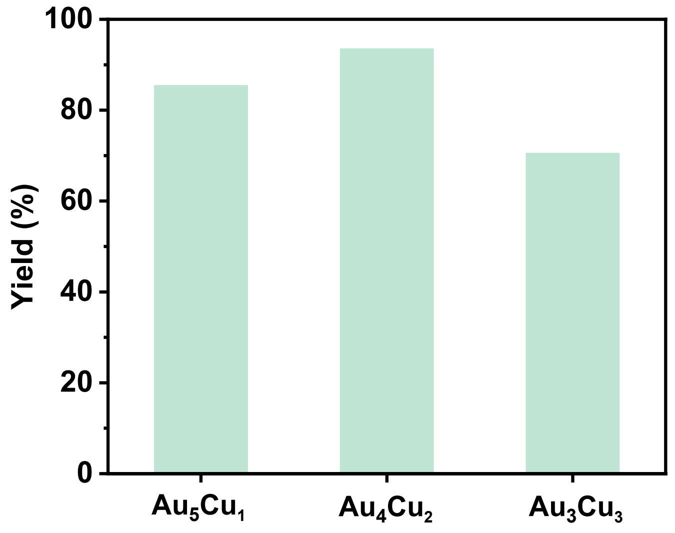

Supplementary Fig. 13. PGA yields of different electrocatalysts at 0.7 V vs. RHE.

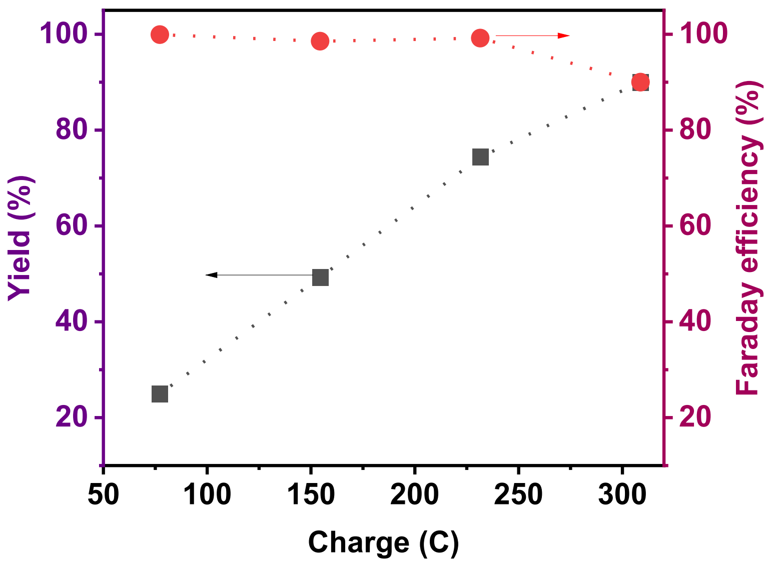

Supplementary Fig. 14. PGA yields and FEs of Au_4Cu_2 under different passing charges at 1.0 V vs. RHE.

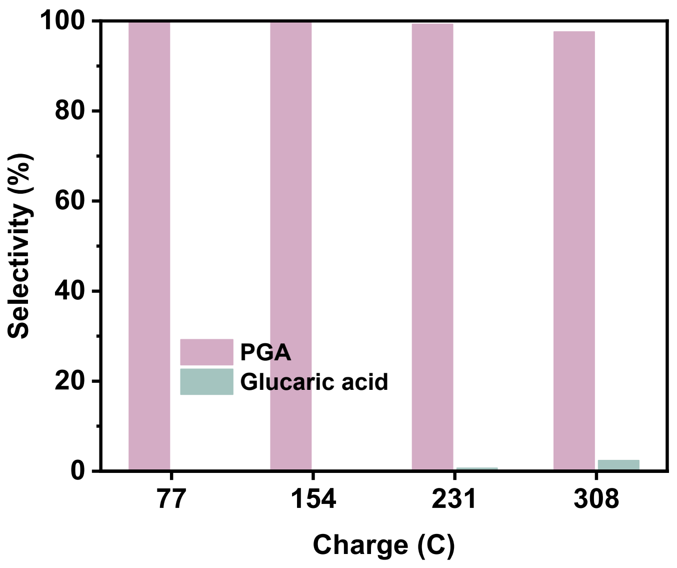

Supplementary Fig. 15. Products selectivity of Au_4Cu_2 toward GOR at 0.7 V vs. RHE.

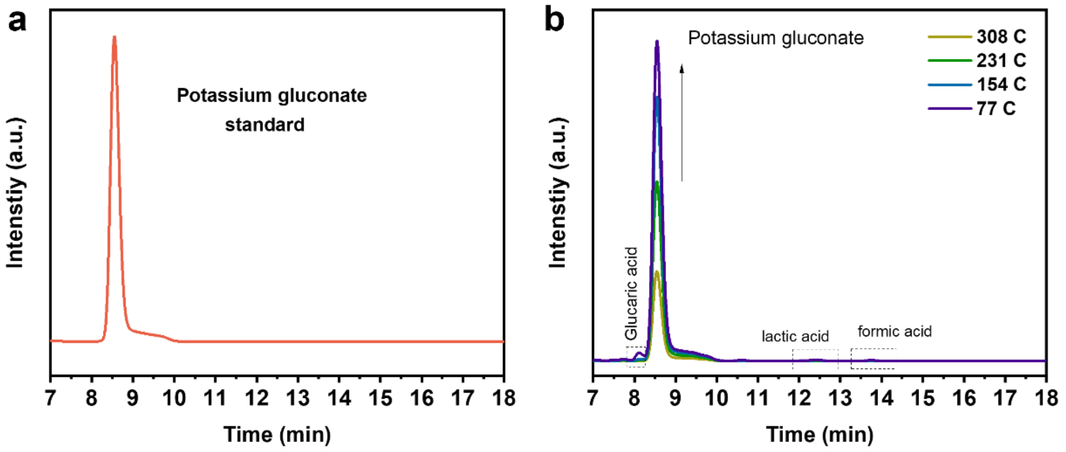

Supplementary Fig. 16. The HPLC spectra of (a) potassium gluconate standard, and (b) potassium gluconate products during GOR.

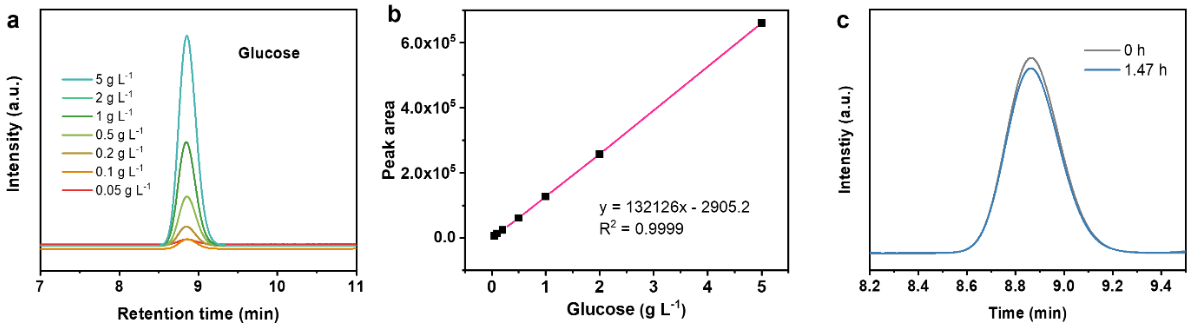

Supplementary Fig. 17. (a) HPLC spectra of glucose detected by differential refractive index detector, and (b) the corresponding calibration curves, (c) HPLC spectra of glucose degradation under the experimental conditions of GOR.

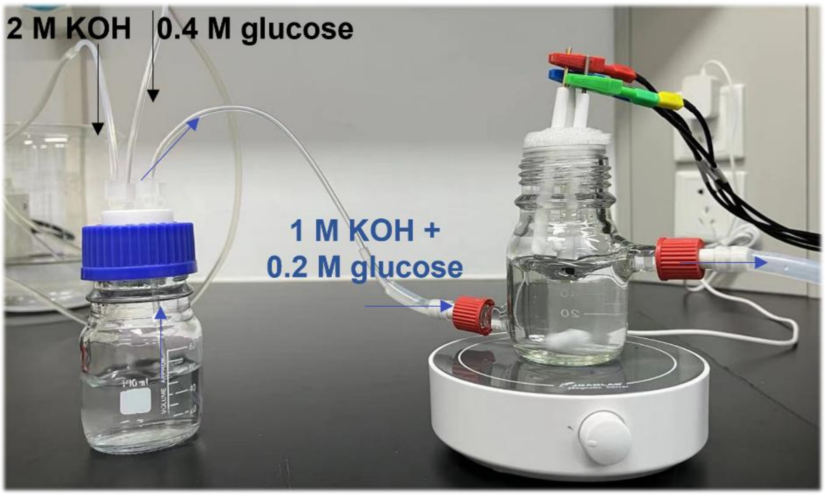

Supplementary Fig. 18. A home-made reactor for glucose electrooxidation under stirring, with continuous supply of flowing electrolyte.

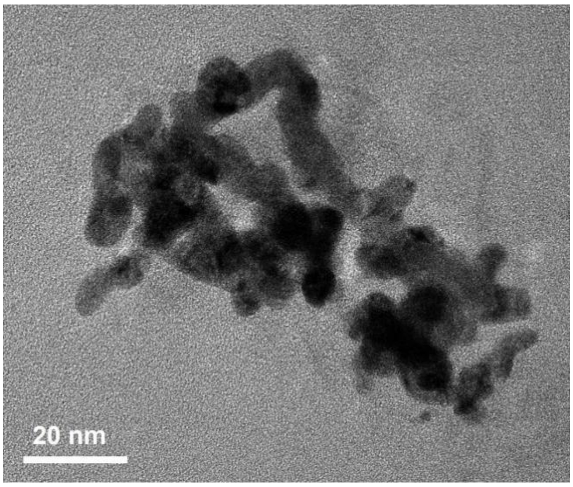

Supplementary Fig. 19. TEM image of Au_4Cu_2 after GOR at 1.0 V vs. RHE.

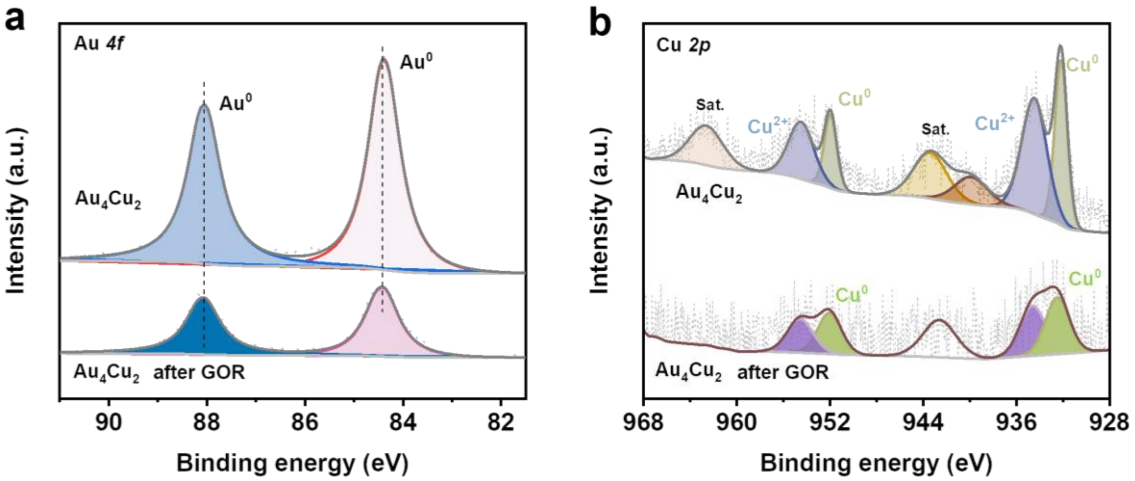

Supplementary Fig. 20. XPS spectra of Au_4Cu_2 before and after GOR at 1.0 V vs. RHE: (a) Au *4f*, (b) Cu *2p*.

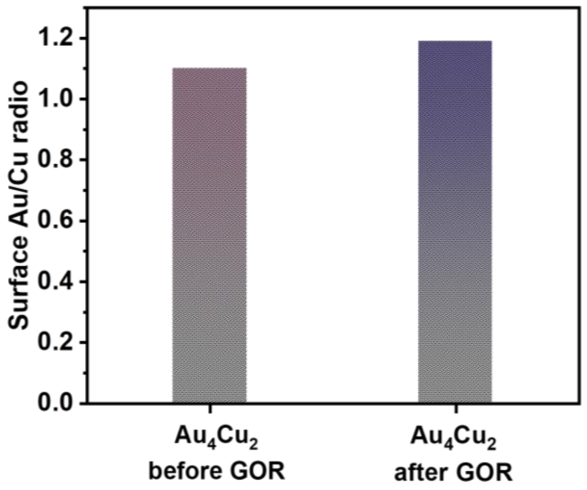

Supplementary Fig. 21. Surface Au/Cu radio of Au_4Cu_2 catalyst before and after GOR obtained from TOF-SIMS.

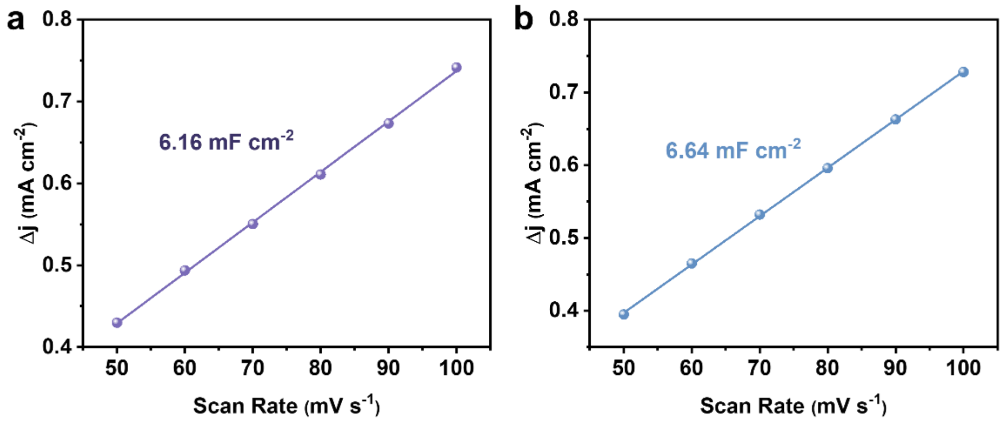

Supplementary Fig. 22. The extracted double-layer capacitances (C_{dl}) of Au_4Cu_2 (a) before and (b) after GOR.

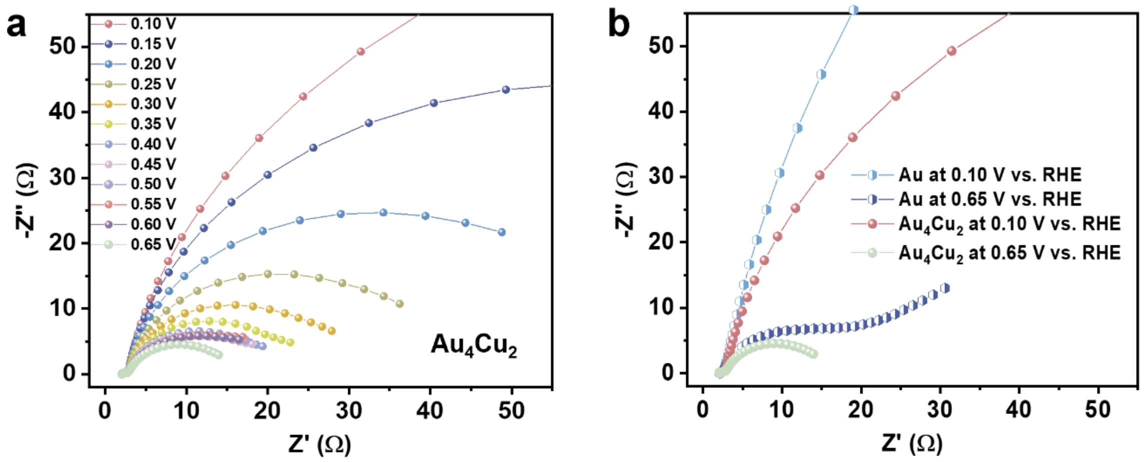

Supplementary Fig. 23. In situ EIS of (a) Au_4Cu_2 in 1 M KOH with 0.2 M glucose, (b) EIS of Au_4Cu_2 and Au at different potentials.

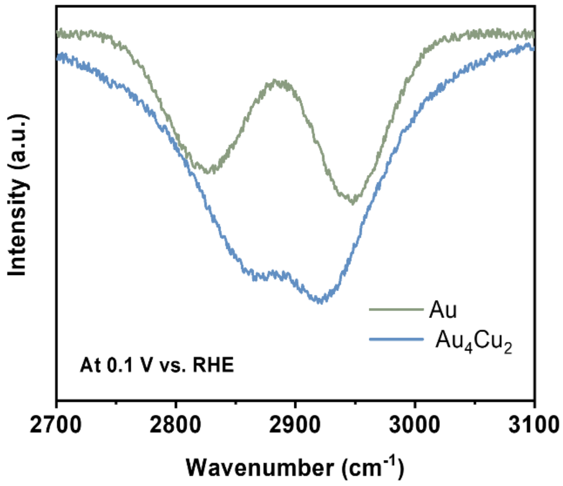

Supplementary Fig. 24. FTIR spectra of (e) Au_4Cu_2 and (f) Au at 0.1 V vs. RHE during GOR.

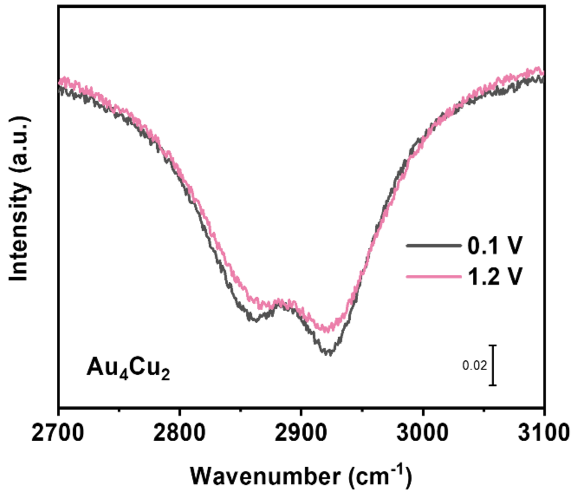

Supplementary Fig. 25. FTIR spectra of Au_4Cu_2 at 0.1 V and 1.2 V vs. RHE during GOR.

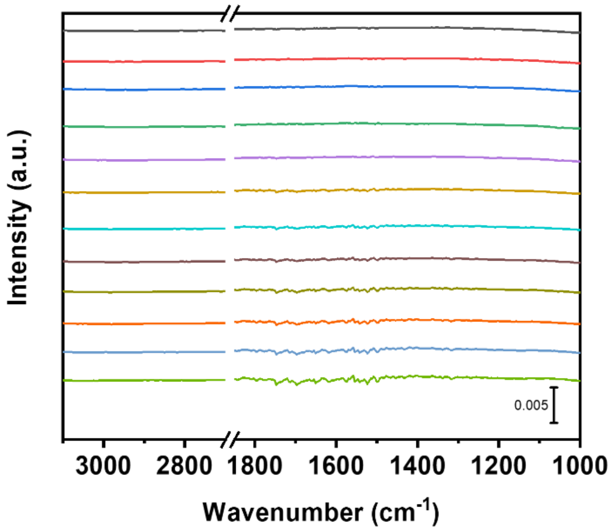

Supplementary Fig. 26. In situ electrochemical FTIR spectra of Cu at different potentials for GOR in 1 M KOH with 0.2 M glucose (from 0.1 to 1.2 V vs. RHE).

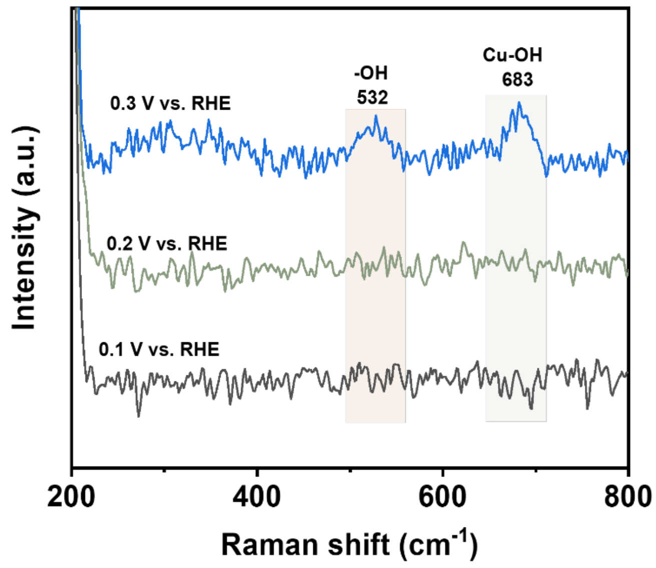

Supplementary Fig. 27. Raman spectra of the Au_4Cu_2 alloy in 1 M KOH.

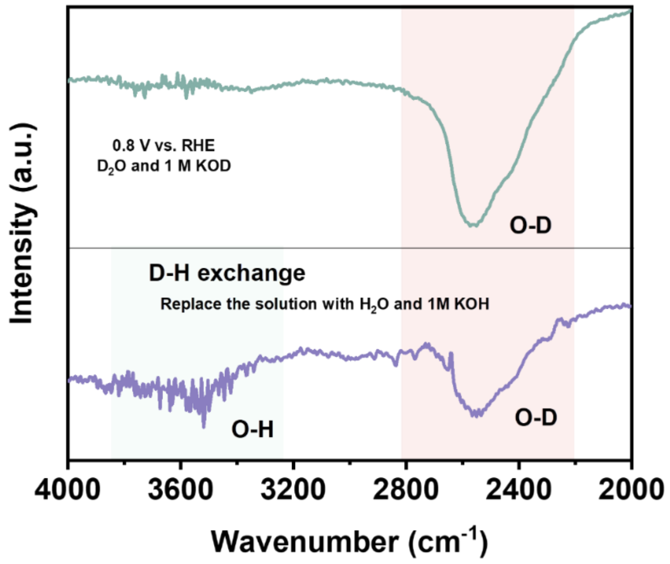

Supplementary Fig. 28. H-D exchange FTIR experiment over Au_4Cu_2.

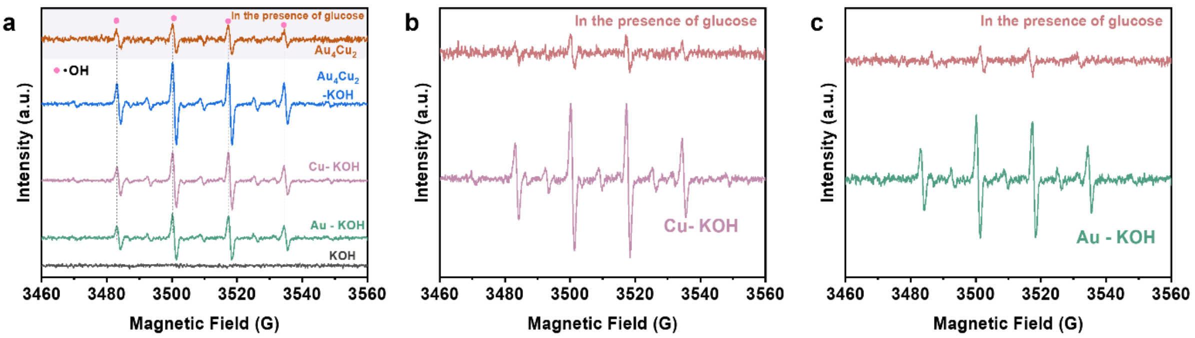

Supplementary Fig. 29. EPR spectra under different test conditions.

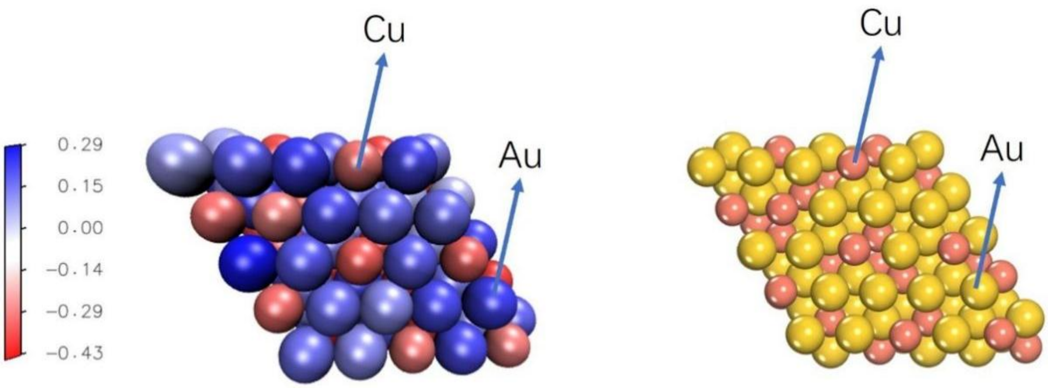

Supplementary Fig. 30. Bader charge and the corresponding model of Au_4Cu_2.

The positive value of Au indicates that the Au atoms accept electrons from Cu atoms, while the negative value of Cu indicates the transfer of electrons from Cu atoms to the Au atoms.

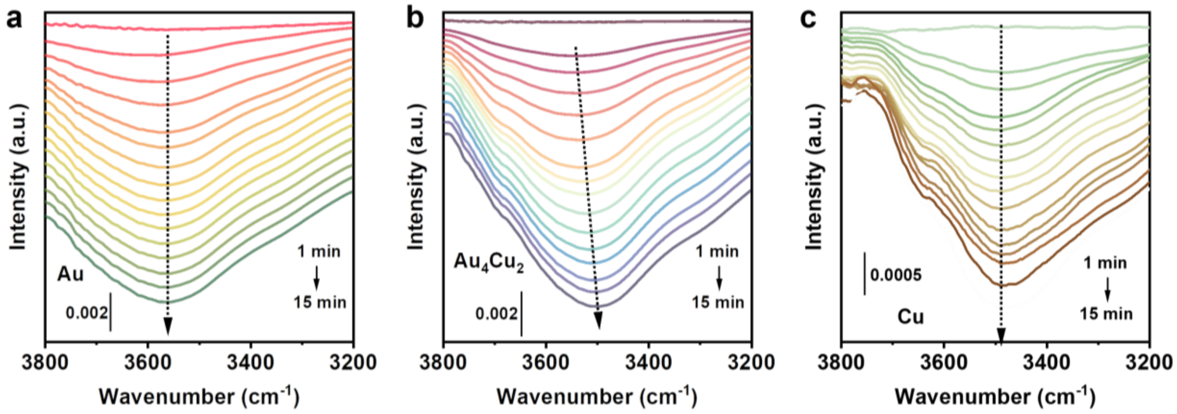

Supplementary Fig. 31. In situ IRAS spectra of (a) Au, (b) Au_4Cu_2, and (c) Cu at 1.0 V vs. RHE.

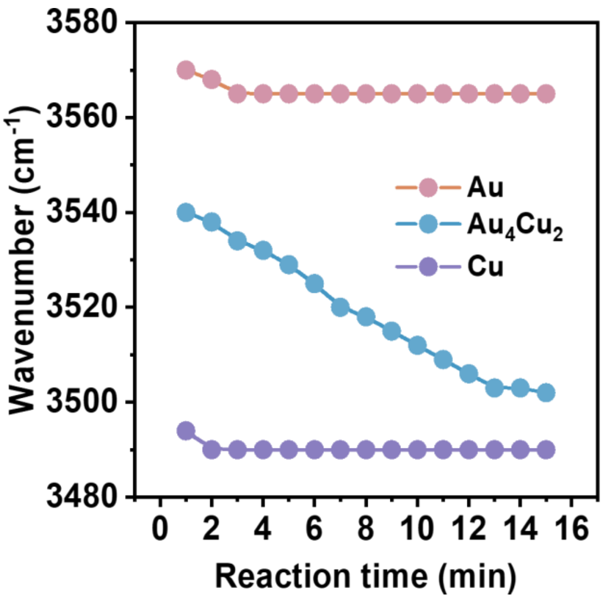

Supplementary Fig. 32. Changes of peak position *OH from in situ IRAS spectra.

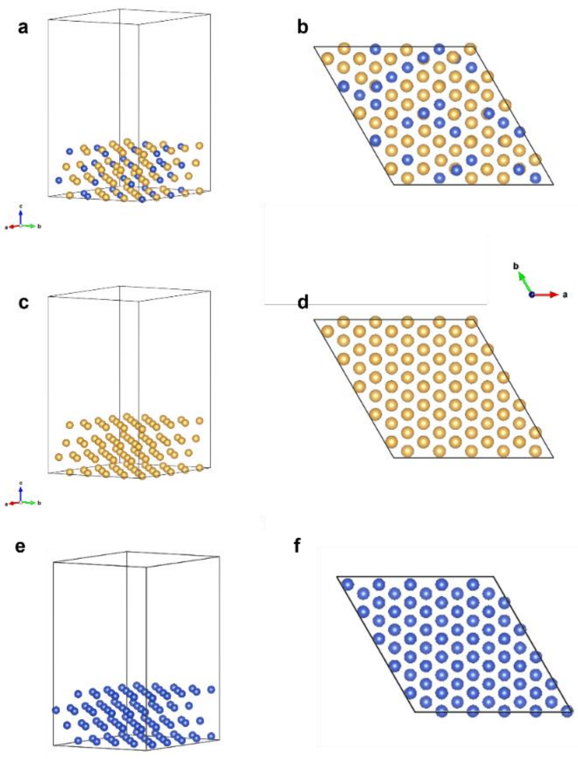

Supplementary Fig. 33. Optimized models of (a, b) Au_4Cu_2 (111), (c, d) Au (111), and (e, f) Cu (111)

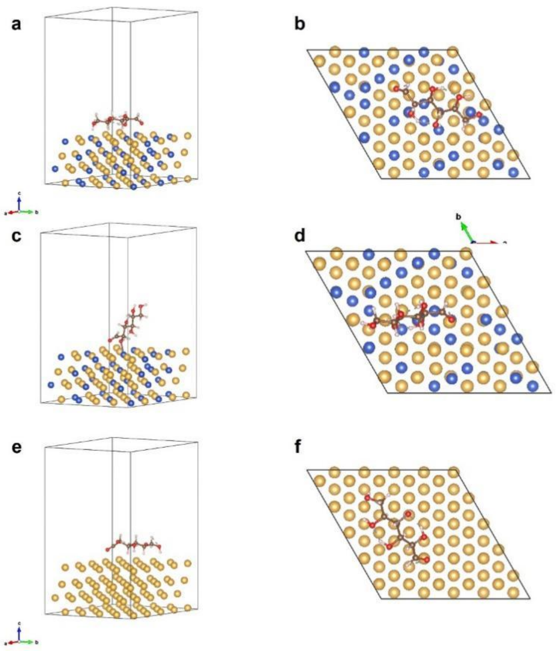

Supplementary Fig. 34. Optimized models of *glucose on (a, b) Au site of Au_4Cu_2 (111), (c, d) Cu site of Au_4Cu_2 (111), and (e, f) Au (111).

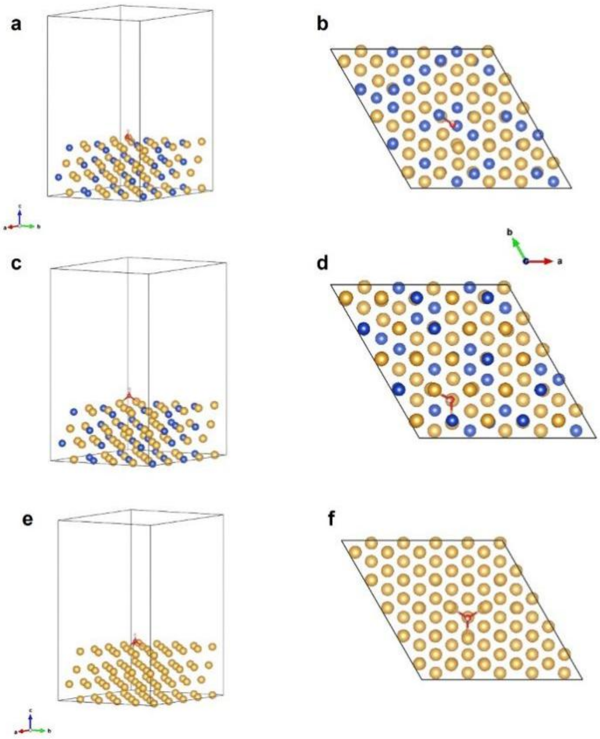

Supplementary Fig. 35. Optimized models of *OH on (a, b) Cu site of Au_4Cu_2 (111), (c, d) Au site of Au_4Cu_2 (111), and (e, f) Au (111).

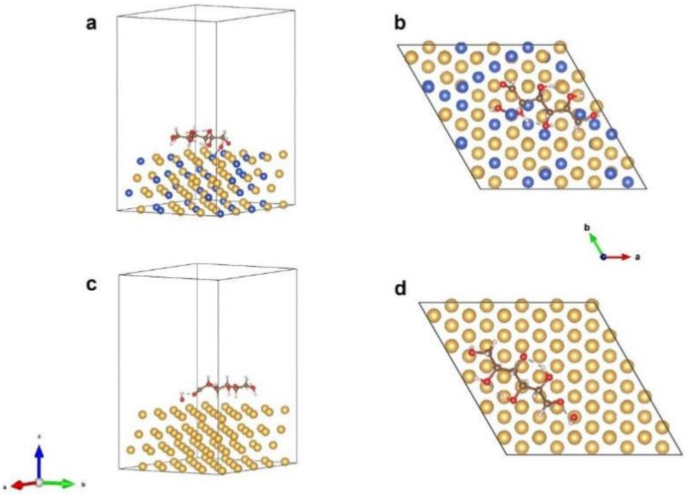

Supplementary Fig. 36. Optimized models of *glucose and *OH on (a, b) Au_4Cu_2 (111), and (c, d) Au (111).

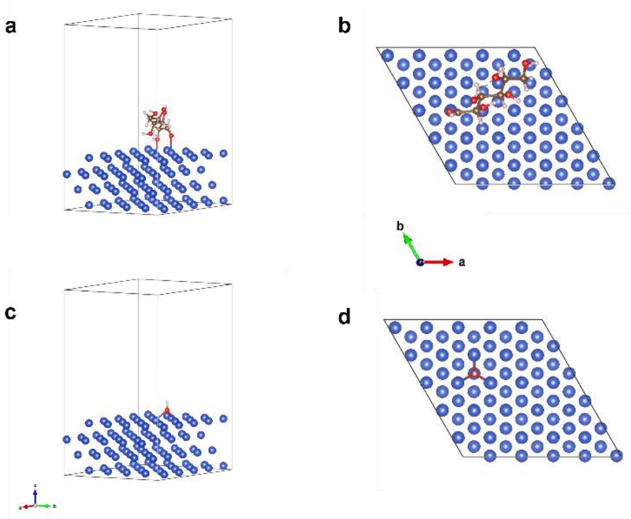

Supplementary Fig. 37. Optimized models of (a, b) *glucose on Cu (111), and (c, d) *OH on Cu (111).

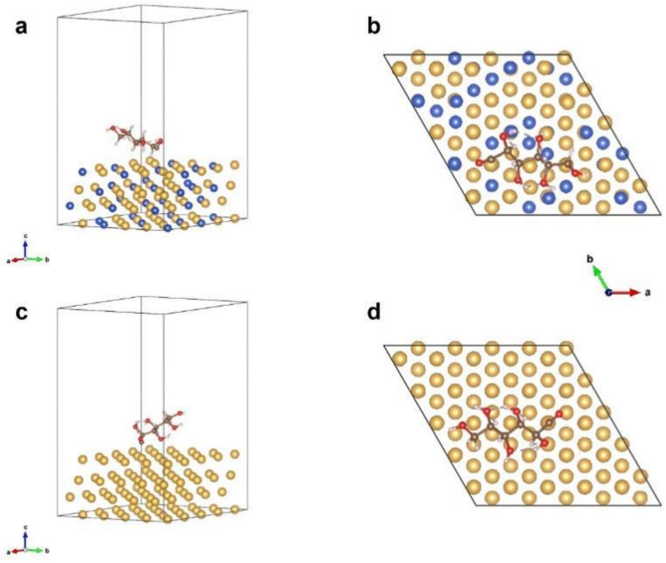

Supplementary Fig. 38. Optimized models of *CO-R on (a, b) Au_4Cu_2 (111), and (c, d) Au (111).

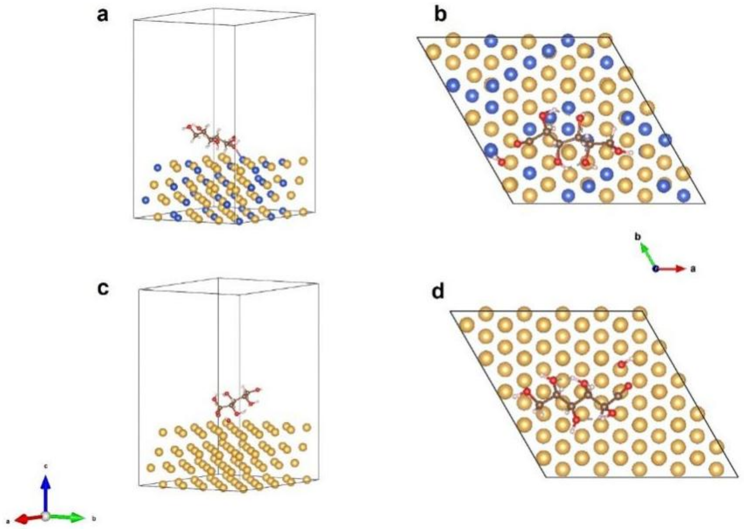

Supplementary Fig. 39. Optimized models of *CO-R and *OH on (a, b) Au_4Cu_2 (111), and (c, d) Au (111).

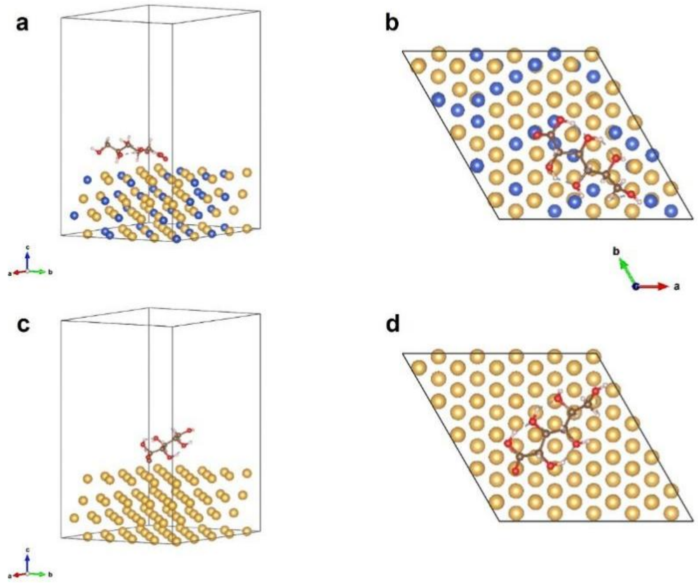

Supplementary Fig. 40. Optimized models of *COOH-R on (a, b) Au_4Cu_2 (111), and (c, d) Au (111).

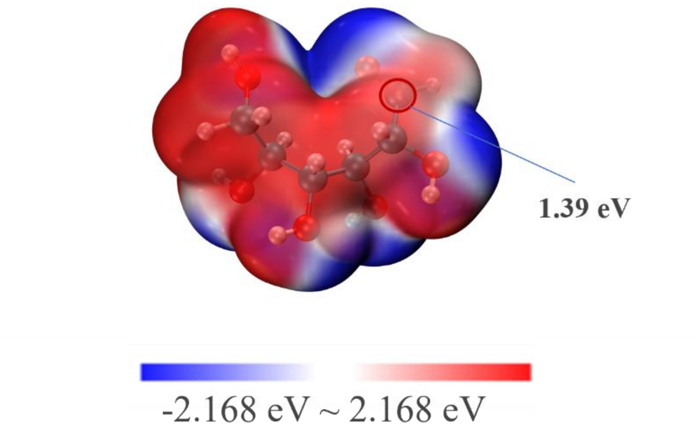

Supplementary Fig. 41. The electrostatic potential distributions of glucose, where blue regions indicate electron-rich domain, and red regions denote electron-lean domain.

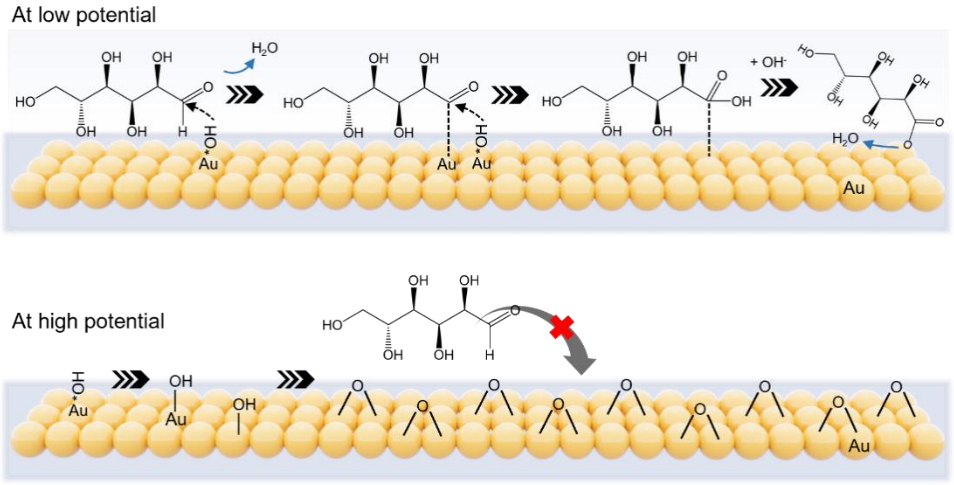

Supplementary Fig. 42. Schematic diagrams of chemical changes on Au surface in 0.2 M glucose with 0.1 M KOH at low and high potentials.

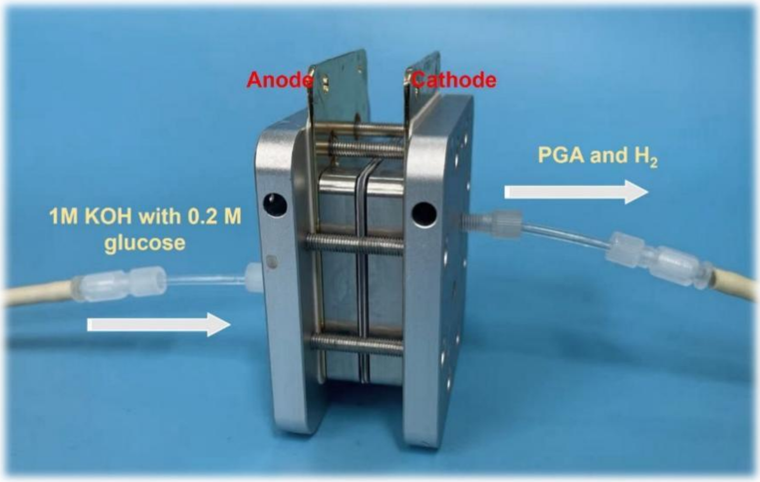

Supplementary Fig. 43. Digital photos of the membrane-free flow cell system.

The anode and cathode share a liquid circulation driven by a peristaltic pump. The flow velocity is set to 100 ml min^{-1} ensuring continuous glucose supply and rapid removal of the products from the electrode surfaces.

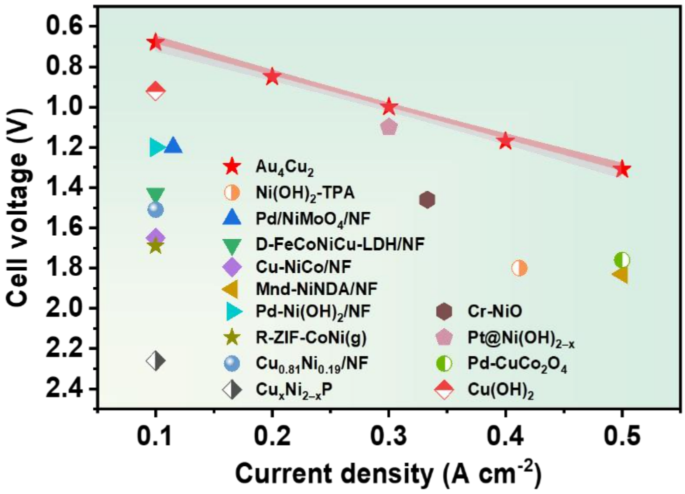

Supplementary Fig. 44. Comparison of coupled system performance at different current densities.

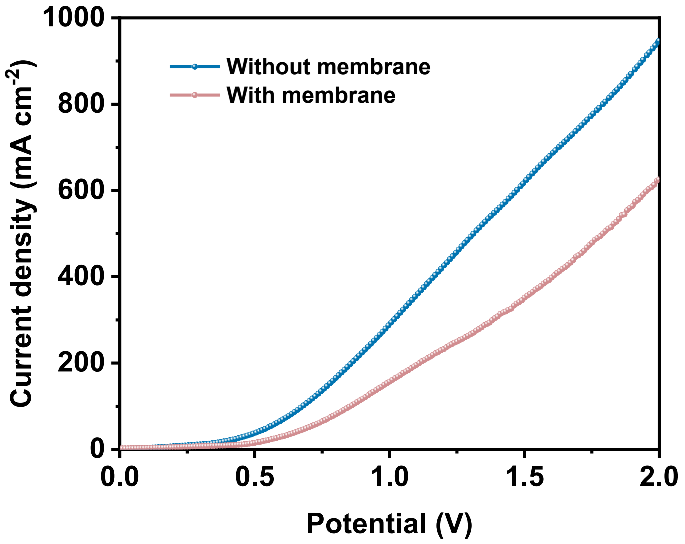

Supplementary Fig. 45. LSV curves of flow cell with membrane and with membrane using Au_4Cu_2 alloy as anode and cathode.

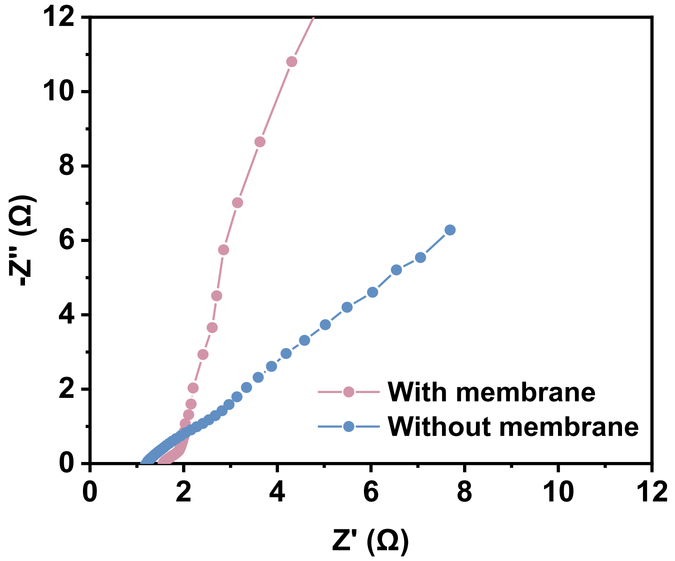

Supplementary Fig. 46. EIS of flow cell with membrane and with membrane using Au_4Cu_2 alloy as anode and cathode.

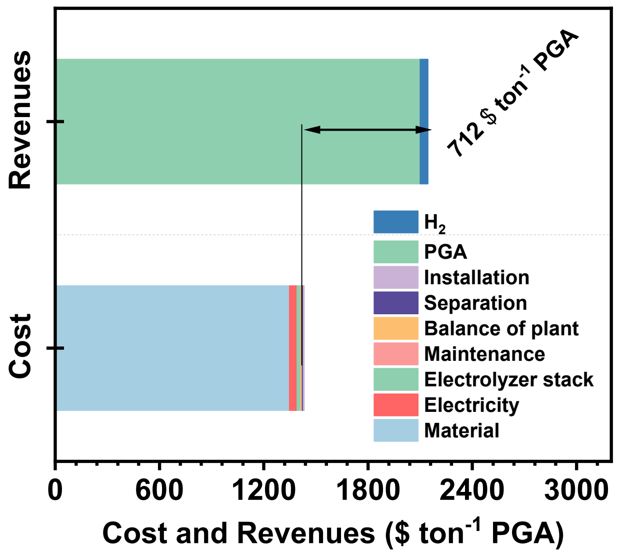

Supplementary Fig. 47. TEA results at 500 mAcm^{-1}.

Supplementary Tables

Supplementary Table 1 The element measurements of post-reaction electrolyte by ICP-OES.

Sample	ICP-OES Analysis (at.%)		Calculate composition from Vegard's law	
	Au	Cu	Au	Cu
Au_5Cu_1	83.49	16.51	88.68	11.32
Au_4Cu_2	67.96	32.04	80.11	19.89
Au_3Cu_3	54.66	45.34	69.64	30.36
Au_4Cu_2 after stability tests	68.27	31.73	-	-

Supplementary Table 2 Performances of recently reported catalysts for biomass electrochemical reforming in alkaline condition.

Electrocatalyst	Electrolyte	Feedstock	Current density (mA cm^{-2})	Potential (V_{RHE})	Ref
Au_4Cu_2	1 M KOH + 0.2 M glucose	potassium gluconate	10<br>100<br>500<br>1000	0.14<br>0.32<br>0.74<br>1.40	This Work
CNTs@Co/CoP	1 M KOH + 0.5 M Glucose	Gluconic acid	10	1.42	1
Pd/$NiMoO_4$/NF	1 M NaOH + 1 M Ethylene glycol	sodium glycolate	100	0.79	2
Pd-$Ni(OH)_2$	1 M NaOH + 1 M Ethylene glycol	glycolic acid	100<br>500	0.69<br>1.07	3
Co@NCNT/CW	1 M KOH + 0.1 M xylose	Formate	100	1.36	4
$NiFeO_x$	1 M KOH + 0.1 M glucose	Glucaric acid	200	1.48	5
Co_3FeP_x	1 M KOH + 0.1 M glucose	/	200	1.74	6
hp-PtAu/NF	1 M KOH + 0.5 M glycerol	Lactate	10<br>100	0.26<br>0.57	7
$NiFe_2O_4$/NF	1 M KOH + 0.5 M methanol	Formate	100	1.82	8
$Cu(OH)_2/Cu_2O$	1 M KOH + 0.1 M glucose	Glucaric acid	100	0.92	9
Au/$Ni(OH)_2$	3 M KOH + 0.3 M glycerol	Lactic acid	420	1.00	10
$Pd_{67}Ag_{33}$	1 M NaOH + 1 M Ethylene glycol	glycolic acid	10	0.53	11
NiVRu-LDHs NAs/NF	1 M KOH + 0.1 M glycerol	Formate	150	1.35	12
Cu-NiCo/NF	1 M KOH + 0.1 M Glycerol	Formate	10<br>100	1.23<br>1.33	13
Pd-$CuCo_2O_4$	1 M NaOH + 0.5 m NaCl + 0.3 M EG	Glycolic acid	350	1.07	14

Supplementary Table 3 Comparison of the required cell voltage of our system with various electrocatalysts for H_2 production in alkaline condition

Electrocatalysts	Voltage (V)	Current density (mA cm^{-2})	Ref.
Au_4Cu_2 (+) // Au_4Cu_2 (-)	0.68	100	This Work
	1.31	500	
	2.07	1000	
$Ni(OH)_2$-TPA (+) || Ni-Mo/NF (-)	1.8	412	15
Pd/$NiMoO_4$/NF (+) || NF (-)	1.2	115	2
D-FeCoNiCu-LDH/NF (+) // FeCoNiCuCr-LDH/NF (-)	1.43	100	16
Cu-NiCo/NF (+) || Cu-NiCo/NF (-)	1.65	100	13
Mnd-NiNDA/NF (+) || Pt/C (−)	1.83	500	17
NiFeOx-NF(+) // NiFeNx-NF (–)	*1.38	*100	5
Pd $Ni(OH)_2$/NF (+) || Ni_2P/NF (-)	1.2	100	3
Cr-NiO (+) || Cr-Ni3N (-)	1.46	333	18
NiCo hydroxide (+) || NiCo hydroxide (-)	*1.58	*100	19
$Cu(OH)_2$ (+) || Pt/C (−)	*0.92	*100	9
R-ZIF-CoNi(g) (+) || Pt/C (−)	1.69	100	20
Pt@$Ni(OH)_{2-x}$ (+) || Pt@$Ni(OH)_{2-x}$ (−)	1.1	300	21
NiFeOx-NF (+) || NiFeNx-NF (−)	*1.4	*100	22
$Cu_xNi_{2-x}P$ (+) || Ni foam	2.26	100	23
$Cu_{0.81}Ni_{0.19}$/NF (+) || $Cu_{0.81}Ni_{0.19}$/NF (-)	1.51	100	24
Pd-$CuCo_2O_4$ (+) || CoP (-)	0.87	100	14
	1.76	500	

Note. *The data were obtained through the H-cell or single cell, and others were from the flow cell measurement.

Techno-economic analysis of this electro-reforming process

Supplementary Table 4 Economic parameters and assumptions of chemicals from electro-reforming process.

Chemicals	Price	Source
Glucose [a]	400 \$ ton^{-1}	https://rawchem2021.en.made-in-china.com/product/VnwYEJrbqDcg/China-Purity-99-Glucose-Anhydrous-Powder.html?pv_id=1ilod99qed81&faw_id=1iloda32s9d9
KOH [b]	800 \$ ton^{-1}	https://farmasinochems.en.made-in-china.com/product/kdBfQcNGbVTl/China-High-Purity-Potassium-Hydroxide-KOH-Flakes-90-CAS-1310-58-3.html?pv_id =1ilod7a8m85d&faw_id=1ilod7m7952
Water	0.22 \$ ton^{-1}	Guangzhou, China
Potassium gluconate	2100 \$ ton^{-1}	https://dc51b833077ec00f.en.made-in-china.com/product/GdoTNvfAXhpb/China-China-Factory-Specializing-in-Wholesale-Potassium-Gluconate-299-27-4.html?pv_id=1ilod5fli6f8&faw_id=1ilod6b3qe23
water	0.31 \$ ton^{-1}	Guangzhou, China (Conghua)
H_2	0.41 \$ Nm^{-3}	https://hq.smm.cn/h5/hydrogen-price-chart

The calculation process:

For an industrial-related scenario, the production of PGA can be assumed to be 1ton day^{-1}, the yield and the Faradaic efficiency of PGA is assumed to be 94%, and the Faradaic efficiency of H_2 is assumed to be 100%. The electrolyte consists of 1.0 M KOH and 200 mM glucose.

1. Material costs

Glucose imput = 1 ton day^{-1} ÷ M_{PGA} × $M_{glucose}$ ÷ Yield = 1 ton day^{-1} ÷ 234 × 180 ÷ 94% = 0.818 ton day^{-1}

glucose cost = 0.818 × 400 = 327.2 \$ day^{-1}

Mass of glucose needed in 1.0 L of H_2O = 1.0 L × 0.2 mol L^{-1} × 180 g mol^{-1} = 36 g

H_2O consumption = 0.818 × 10^{6} ÷ 36 = 22722 L day^{-1}

H_2O cost = 22722 × 10^{-3} × 0.31 = 7.04 \$ day^{-1}

KOH consumption = 22722 × 1.0 × 56 × 10^{-6} = 1.27 ton day^{-1}

KOH cost = 1.27 × 800 = 1016 \$ day^{-1}

Total current = 0.818 ton day^{-1} × 10^{6} ÷ 180 × 2 × 96,485 ÷ (24 × 3,600) ÷ 94% = 10798 A

day^{-1}

Power = UI = 1.4 V × 10798 A × 10^{-3} = 15.12 kW

Electricity cost =15.12 kW × 24 h × x \$ kWh^{-1} = 362.9 x \$ day^{-1} (x refers to the electricity price)

Electrolyzer area = 10798/y cm^2 here, y refers to the current density (A cm^{-2})

H_2 output = 10798 × 24 × 3,600 × 2 × 10^{-3} ÷ (2 × 96,485) = 9.67 kg day^{-1}

2. Capital cost

For 1 m^2 catalyst, the mass of Au is 78.8 g, and the mass of Cu is 12.7 g. The price of Au is 93.67 \$ g^{-1}, and the price of Cu is 0.12 \$ g^{-1}.

Catalyst cost = 78.8 × 93.67 + 12.7 × 0.12 = 7382.7 \$ per m^2

The total cost of the electrolyzer is assumed to be 10,000 \$ per m^2.

The capital cost of cell is 1.74 \$ per cm^2. The electrolyzer is assumed to run for 5 years (running 350 days $year^{-1}$)

Electrolyzer stack cost = 10798/y × 1.74 / (5×350) = 10.74/y \$ day^{-1}

Installation cost: Electrolyzer stack cost × Lange coefficient (0.2) = 2.15/y \$ day^{-1}

Maintenance cost = Capital cost × 5% = 0.54/y \$ day^{-1}

In general, Balance of plant (BOP) is 35% of the electrolyzer stack cost.

BOP cost = 10.74/y × 35% = 3.76/y \$ day^{-1}

Separation capital cost = 362.9 x × 30% = 108.9 x \$ day^{-1}

3. Total cost

Total cost of PGA per day = Glucose cost + H_2O cost + KOH cost + Electricity cost + Electrolyzer stack cost + Maintenance cost + BOP cost + Separation capital cost + Installation cost = 327.2 + 7.04 + 1016 + 362.9x + 10.74/y + 0.54/y + 3.76/y + 108.9 x + 2.15/y = 471.8x + 1348.24 + 17.16/y

Supplementary references

1. Zhang, Y. *et al.* Core-corona Co/CoP clusters strung on carbon nanotubes as a Schottky catalyst for glucose oxidation assisted H_2 production. *J. Mater. Chem. A* **9**, 10893-10908 (2021).
2. Shi, K., Si, D., Teng, X., Chen, L. & Shi, J. Pd/$NiMoO_4$/NF electrocatalysts for the efficient and ultra-stable synthesis and electrolyte-assisted extraction of glycolate. *Nat. Commun.* **15**, 2899 (2024).
3. Liu, F. *et al.* Concerted and selective electrooxidation of polyethylene-terephthalate-derived alcohol to glycolic acid at an industry-level current density over a Pd-$Ni(OH)_2$ catalyst. *Angew. Chem. Int. Ed.* **62**, e202300094 (2023).
4. Li, D. *et al.* Wood-derived, monolithic chainmail electrocatalyst for biomass-assisted hydrogen production. *Adv. Energy Mater.* **13**, 2300427 (2023).
5. Liu, W.-J. *et al.* Efficient electrochemical production of glucaric acid and H_2 via glucose electrolysis. *Nat. Commun.* **11**, 265 (2020).
6. Miao, J. *et al.* "Carbohydrate-universal" electrolyzer for energy-saving hydrogen production with Co_3FeP_x@NF as bifunctional electrocatalysts. *Appl. Catal. B Environ.* **263**, 118109 (2020).
7. Li, Y. *et al.* PtAu alloying-modulated hydroxyl and substrate adsorption for glycerol electrooxidation to C3 products. *Energy Environ. Sci.* **17**, 4205-4215 (2024).
8. Du, X. *et al.* Highly efficient and robust nickel-iron bifunctional catalyst coupling selective methanol oxidation and freshwater/seawater hydrogen evolution via CO-free pathway. *Chem. Eng. J* **452**, 139404 (2023).
9. Zhang, Y. *et al.* Coupling glucose-assisted Cu(I)/Cu(II) redox with electrochemical hydrogen production. *Adv. Mater.* **33**, 2104791 (2021).
10. Yan, Y. *et al.* Electrocatalytic upcycling of biomass and plastic wastes to biodegradable polymer monomers and hydrogen fuel at high current densities. *J. Am. Chem. Soc.* **145**, 6144-6155 (2023).
11. Chen, J. *et al.* Unveiling synergy of strain and ligand effects in metallic aerogel for electrocatalytic polyethylene terephthalate upcycling. *Proc. Natl. Acad. Sci. U.S.A.* **121**,

e2318853121 (2024).

12. Qian, Q. *et al.* Electrochemical biomass upgrading coupled with hydrogen production under industrial-level current density. *Adv. Mater.* **35**, 2300935 (2023).

13. Li, C. *et al.* Efficient electrocatalytic oxidation of glycerol to formate coupled with nitrate reduction over Cu-doped NiCo alloy supported on nickel foam. *Angew. Chem. Int. Ed.* **63**, e202411542 (2024).

14. Liu, K. *et al.* Energy-saving hydrogen production by seawater splitting coupled with PET plastic upcycling. *Adv. Energy Mater.* **14**, 2304065 (2024).

15. Chen, D., Li, W., Liu, J. & Sun, L. Bio-inspired proton relay for promoting continuous 5-hydroxymethylfurfural electrooxidation in a flowing system. *Energy Environ. Sci.* **18**, 3120 (2025).

16. Wu, X. *et al.* Multi-site catalysis of high-entropy hydroxides for sustainable electrooxidation of glucose to glucaric acid. *Energy Environ. Sci.* **17**, 3042 (2024).

17. Xiong, D. *et al.* Manipulating dual-metal catalytic activities toward organic upgrading in upcycling plastic wastes with inhibited oxygen evolution. *ACS Nano* **18**, 20340-20352 (2024).

18. Li, S. *et al.* Chromium-doped nickel oxide and nickel nitride mediate selective electrocatalytic oxidation of sterol intermediates coupled with H_2 evolution. *Angew. Chem. Int. Ed.* **62**, e202306553 (2023).

19. He, Z. *et al.* Promoting biomass electrooxidation via modulating proton and oxygen anion deintercalation in hydroxide. *Nat. Commun.* **13**, 3777 (2022).

20. Shen, Y. *et al.* Promoted electrochemical reconstruction of glassy metal–organic frameworks for efficient electrocatalytic 5-hydroxymethylfurfural oxidation. *Adv. Energy Mater.*, **15**, 2405364 (2024).

21. Ma, F. *et al.* Selectively steering the retention and cleavage of C–C bond in electrooxidation of PET plastic and biomass-derived alcohols by defective $NiOH_{2-x}$-Supported Pt. *ACS Catal.* **15**, 4759-4769 (2025).

22. Liu, W. J. *et al.* Efficient electrochemical oroduction of glucaric acid and H_2 via glucose electrolysis. *Nat. Commun.* **11**, 265 (2020).

23. Ma, L. *et al.* Promoting electrocatalytic glycerol C─C bond cleavage to formate coupled with H_2 production over a $Cu_xNi_{2-x}P$ catalyst. *Adv. Energy Mater.* **14**, 2401061 (2024).
24. Ren, X. *et al.* Nickel-copper alloying arrays realizing efficient co-electrosynthesis of adipic acid and hydrogen. *J. Energy Chem.* **101**, 7-15 (2025).
