# Supplementary material for: Regulating adsorption selectivity by charge-polarized Auδ−-Cuδ+ site for stable glucose electrooxidation
Source: Nat Commun. 2026 Apr 23;17:4372. doi: 10.1038/s41467-026-72465-x (PMC13176337; doi:10.1038/s41467-026-72465-x)
Supplement: Supplementary file 2 — Description of Additional Supplementary Files [file 41467_2026_72465_MOESM2_ESM.pdf]

Description of Additional Supplementary Files

File Name: Supplementary Data 1

Description: The optimized computational model CIF files for Au_4Cu_2, Au and Cu (Supplementary Figs. 33-40) are provided.
